# Adrenal Cortex-Sparing Surgery for Bilateral Multiple Pheochromocytomas in a Patient with Von Hippel-Lindau Disease

**DOI:** 10.1155/2012/659104

**Published:** 2012-10-10

**Authors:** Tarık Esen, Ömer Acar, Ahmet Tefekli, Ahmet Musaoğlu, İzzet Rozanes, Ali Emre

**Affiliations:** ^1^School of Medicine, Koç University, 34450 Istanbul, Turkey; ^2^Department of Urology, VKF American Hospital, 34365 Istanbul, Turkey; ^3^Department of Radiology, VKF American Hospital, 34365 Istanbul, Turkey; ^4^Department of General Surgery, VKF American Hospital, 34365 Istanbul, Turkey

## Abstract

Pheochromocytomas can be a part of familial neoplastic syndromes, in which case they tend to be multiple and involve both adrenal glands. Therefore, sparing adrenocortical function represents a major concern while dealing with these hereditary lesions. Herein, we describe the clinical characteristics and the management strategy of a patient with von Hippel-Lindau (VHL) disease who had multiple, bilateral pheochromocytomas as well as bilateral renal masses, pancreatic masses, and a paracaval mass. Only a portion of the left adrenal gland has remained in situ after two consecutive open surgeries and a percutaneous radiofrequency ablation which have been performed to treat the various components of this syndrome. No adrenal or extra-adrenal pheochromocytoma recurrences have been detected during a follow-up period of more than 2 years. Pancreatic and adrenal functions were normal throughout the postoperative period and never necessitated any replacement therapy. Adrenal cortex-sparing surgery is a valid option for VHL disease patients who present with synchronous bilateral adrenal pheochromocytomas.

## 1. Introduction 

Adrenal surgery has gained popularity throughout the world most probably because of the increased radiologic detection of small, solid, asymptomatic adrenal masses. These incidentalomas are mostly small in size and nonfunctioning. However, surgery is needed either because of the size or the hormone-producing nature of the lesion, in at least 15% of the cases [1].

Adrenal surgery has been accepted as a total adrenal resection per definition. In a patient with unilateral disease and a healthy contralateral adrenal gland, this is mostly not a problem. Yet the radiologic presence of the contralateral gland is not necessarily an evidence for healthy function. Moreover, adrenal masses are common and mostly bilateral and/or multiple in patients with hereditary syndromes, where a bilateral adrenalectomy could have devastating side effects either with the resulting adrenal insufficiency or the need for life-long hormonal replacement [2].

Recently, the concept of partial adrenalectomy is introduced for the above-mentioned reasons and prompted us to report a case where a staged cortex-sparing adrenal surgery was done in a patient with von Hippel-Lindau (VHL) disease and successfully eliminated multiple bilateral pheochromocytomas without causing adrenal insufficiency.

## 2. Case Report

**Figure 1:**
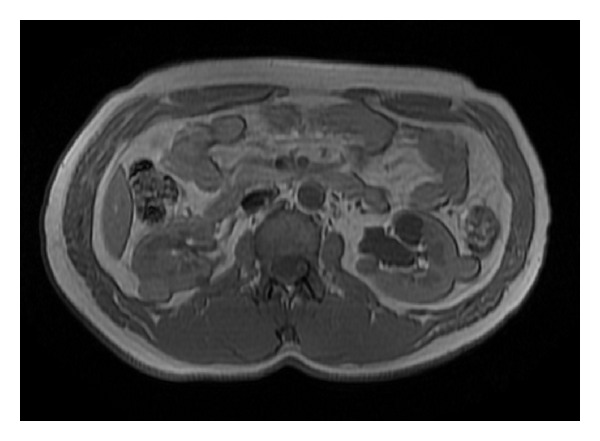
T1-weighted abdominal MRI in the axial plane demonstrating bilateral multiple renal masses of varying sizes and MR signal intensities.

**Figure 2:**
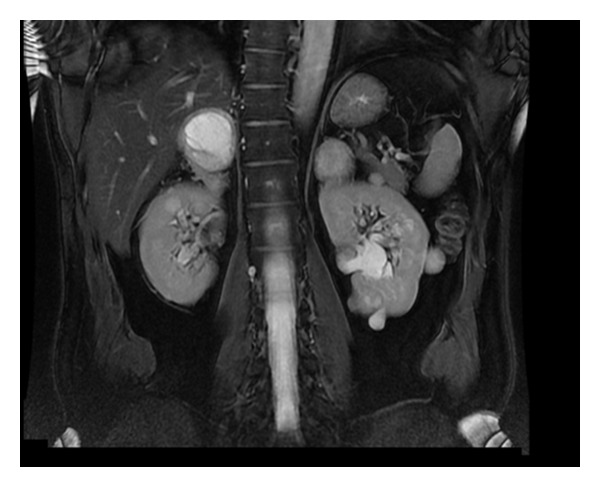
T2-weighted MR image in the coronal plane demonstrating bilateral, adrenal masses and renal cysts. The large adrenal mass on the right side has almost completely replaced the adrenal tissue.

**Figure 3:**
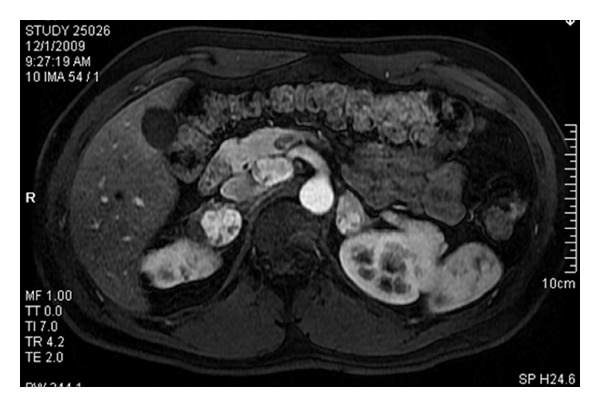
Arterial phase T1-weighted image in the axial plane following intravenous contrast depicts hypervascular adrenal masses on both sides and an approximately 3 cm hypervascular mass in the uncinate process of the pancreas consistent with a neuroendocrine tumor.

A 31-year-old, otherwise healthy, young male patient presented with incidentally detected bilateral small solid renal and adrenal masses, a cystic mass at the head of the pancreas, and a paracaval mass. The patient defined no relevant family history and had no obvious symptoms. Physical examination findings (including serial blood pressure measurements) were within normal limits. Ultrasound (US) and abdominal magnetic resonance imaging (MRI) were performed to understand the nature of a recent, blunt, nonspecific upper abdominal pain and revealed 3 small, solid masses in the right kidney measuring between 1 and 4 cm and 5 masses in the left kidney measuring between 1 and 3 cm in size. All were suggestive of renal cell carcinomas (RCCs) (Figure 1). An 8 cm solid mass completely replaced the right adrenal gland, while there were 5 small masses on the left side, all consistent with pheochromocytomas (Figure 2). The paracaval mass, measuring 4 cm in size, could be not only an enlarged lymph node but also a paraganglioma. The imaging findings of the cystic mass at the uncinate process of pancreas were suggestive of a neuroendocrine tumor (Figure 3). All other radiologic studies, including spinal and cerebral MRI, were normal. Serum and urine biochemical workup (including cortisol and vanilmandelic acid measurements) revealed normal findings regarding the functional status of the adrenal masses.

The patient was diagnosed as VHL disease and a staged surgical approach was recommended based on the radiologic findings. In the first stage, through a subcostal anterior transperitoneal approach, right adrenalectomy, resection of the paracaval mass, and enucleation for the 9 small solid renal masses were performed. Intraoperative frozen section findings were inconclusive about the actual biologic behaviour of the mass located in the uncinate process of the pancreas. Additionally, two other smaller pancreatic masses were palpated during the operation. Given the young age of the patient, we decided to perform a Whipple procedure to remove these pancreatic lesions. Pathologic diagnoses were Fuhrman grade 1, cystic clear-cell renal cell carcinoma (RCC) for only 1 of the renal masses (the rest were reported to be benign nodules), a pheochromocytoma (8 cm in maximal diameter) in the right adrenal completely destroying the gland architecture, a paraganglioma (2 cm in maximal diameter), and benign, well-differentiated neuroendocrine tumors of the pancreas.

**Figure 4:**
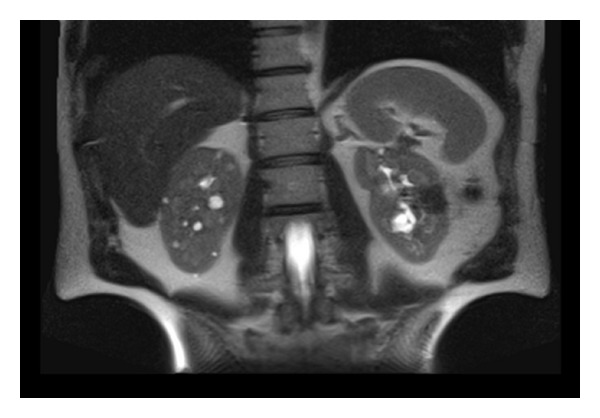
Abdominal MRI following two sessions of open surgery and radiofrequency ablation for a left-sided recurrent solid mass. There is no sign of recurrent or residual solid tumor in the kidneys. Remnant adrenal tissue on the left side was free of any recurrent lesion and was measuring 24.62 × 9.48 mm in diameter. Multiple cysts are visible in the right kidney.

The postoperative period was uneventful so the second stage was performed within 6 weeks. This time, through an intercostal extraperitoneal approach, 5 pheochromocytomas (largest of which was measuring 5 cm in maximal diameter) were removed in cortex-sparing fashion and 4 Fuhrman grade 2, cystic clear-cell RCCs were enucleated. Postoperative course was uneventful without any complications. After a followup of 1,5 years, the patient developed a left 1 cm renal mass, which was histologically proven by ultrasound-guided percutaneous biopsy to be a RCC and was treated by percutaneous, ultrasound-guided radiofrequency ablation. Pancreatic and adrenal functions were normal throughout the follow-up period and never necessitated any replacement therapy. Blood cortisol level (blood sample taken at 8 : 00 in the morning) was 21 *μ*g/dL upon his last control visit. There was no adrenal or extra-adrenal pheochromocytoma recurrence being detected to date (Figure 4). 

## 3. Discussion

Adrenal incidentalomas are unsuspected adrenal masses greater than 1 cm in diameter, identified on cross-sectional imaging performed for unrelated causes. The frequency of adrenal incidentalomas is relatively high, with contemporary imaging series reporting an incidence of approximately 5% [1]. Approximately 20% of adrenal incidentalomas are found to be potential surgical lesions. Generally, large size (≥4 cm), hormonal activity, and radiologic findings suspicious of malignancy are regarded as surgical indications [2]. The probability of discovering a pheochromocytoma during evaluation and treatment process for adrenal incidentalomas is around 5% [3]. 

Pheochromocytoma can develop in association with familial neoplastic syndromes, which typically have autosomal-dominant patterns of inheritance (multiple endocrine neoplasia, VHL disease). VHL disease is an autosomal dominant syndrome manifested by cerebellar and retinal hemangioblastomas; cysts of the pancreas, kidney, and epididymis; epididymal cystadenoma; pheochromocytoma; clear cell RCC. Incidence ranges from 1 in 30,000 to 1 in 50,000 individuals. It has over 95% penetrance by the age of 65 years in affected individuals [4]. The mean age at presentation is 35 to 40 years [5]. Pheochromocytoma occurs in 10% to 17% of affected persons and appears to be confined to specific families [6, 7]. Pheochromocytomas are particularly troublesome entities in the context of VHL disease because of the rapid and unanticipated release of catecholamines. 

In VHL disease, the incidence of bilateral pheochromocytomas ranges from 40% to 80%; however, there is considerable variability regarding the presence of synchronous disease [8, 9]. The treatment of synchronous hereditary pheochromocytoma is highly controversial. Treatment strategies range from routine bilateral adrenalectomy to surveillance of nonfunctioning tumors [10, 11]. Authorities that advocate bilateral adrenalectomy argue that there is a small but real risk of malignancy [12]. Moreover, bilateral adrenalectomy will prevent the occurrence of catastrophic complications from undetected functioning pheochromocytomas. Conversely, Walther et al. advocate a more conservative approach to the management of pheochromocytoma in families with VHL disease [10]. In their series of 64 patients with VHL disease and pheochromocytoma, 12 patients with newly diagnosed pheochromocytomas, but no signs or symptoms, were followed for a median period of 34.5 months with no related morbidity. The authors concluded that nonfunctioning or subclinical disease can be safely followed, especially if quality of life issues mitigate against surgery [10].

In an attempt to preserve adrenal cortical functions and avoid life-long steroid replacement therapy, some centers prefer subtotal adrenalectomy, which requires a small segment of well-vascularized cortical tissue to be left in situ after tumor excision [13–16]. Proponents justify orthotopic adrenal cortex preservation by predictable occurrence of bilateral disease and low incidence of malignancy in hereditary pheochromocytomas. Furthermore, patients with VHL disease often require multiple operations for associated pathologic conditions, which may be more difficult to treat after bilateral adrenalectomy. Moreover, the risk of experiencing at least 1 acute Addisonian crisis approaches 20% after bilateral adrenalectomy [12]. When considering subtotal cortical-sparing adrenalectomy, the avoidance of permanent steroid dependency and potential Addisonian crises must be weighed against the risk of recurrence, which was reported to be in the range of 21–60% [14]. Once subtotal adrenalectomy has been performed, life-long clinical and biochemical surveillance is warranted to detect recurrent disease as early as possible.

The first report of the preservation of adrenocortical function during surgery for bilateral pheochromocytoma was in 1982 [17]. Since then various reports, documenting the feasibility of such conservation, have been published [18–20]. Beside open surgery, partial adrenalectomy can also be performed by laparoscopic and robot-assisted laparoscopic approaches. Janetschek et al. were the first to document the feasibility of laparoscopic partial adrenal resection for pheochromocytoma [21]. Sasagawa et al. replicated the safety and efficacy of this procedure via the retroperitoneoscopic route [22]. In a recent article, Wang et al. presented their data composed of retroperitoneoscopic partial adrenalectomies performed for small (≤1 cm) adrenal tumours. In their series, there were no long-term complications or recurrence of adrenal tumours during a followup of 2–41 months [23]. Recently, Boris et al. reported the results of 10 patients undergoing robot-assisted laparoscopic partial adrenalectomy for removal of 19 adrenal tumors. Of the 19 tumors removed, 17 were pheochromocytoma and 2 were adrenal-cortical hyperplasia. They denied any local recurrence after a median followup of 16.2 months [24]. In a comprehensive literature review, Kaye et al. analyzed the results of 22 partial adrenalectomy series counting for a total of 417 patients. According to their results, 76% of the cases were performed laparoscopically with less than 1% requiring conversion to an open procedure. Moreover, only 3% of the cases developed a recurrent lesion after a mean follow-up duration of 56 months. Considering the results of the studies that reported data on postoperative steroid replacement, only 5.3% (*n* = 7/133) required corticosteroid supplementation on the long term [25].

 It has been reported that 20% of well-perfused adrenal cortical tissue from a single gland is sufficient to maintain an adequate stress response for the individual [15]. Partial adrenalectomy with orthotopic adrenal cortex preservation may specifically be recommended for patients with single adrenal gland, bilateral adrenal tumors, or hereditary adrenal tumors. Being feasible via open and minimally invasive approaches, partial adrenalectomy may also be performed for those with a normal contralateral gland.

Our patient, although having excluded a relevant family history, harbored most of the classical features of VHL disease. He was classified as having Type 2B disease (pheochromocytoma, renal cell cancer, and pancreatic cyst). He had synchronously detected bilateral renal and adrenal lesions. Therefore, he was scheduled for a staged surgical treatment. During the first stage,the right adrenal gland, which was found to be completely replaced by pheochromocytoma, was removed. At the second stage, a total of 5 pheochromocytomas were excised while preserving an adequate amount of adrenocortical tissue. He did not require any hormonal substitution postoperatively and pheochromocytoma did not recur in a follow-up period of 30 months. Moreover, while ablating the right renal mass, which developed during the follow-up period metachronously, the adrenal functional status did not merit any precaution or medical intervention. 

Given the young age of the patient and the propensity to develop recurrent lesions in hereditary neoplastic syndromes, we were able to treat our patient with acceptable oncologic safety and residual adrenocortical function. 

## 4. Conclusions

Adrenal cortex-sparing surgery is a valid option for VHL disease patients who present with synchronous bilateral adrenal pheochromocytomas. Leaving behind a portion of a single gland can be sufficient for reliable adrenocortical functioning without compromising oncologic safety.
